# Readiness for climate change adaptation in the Arctic: a case study from Nunavut, Canada

**DOI:** 10.1007/s10584-017-2071-4

**Published:** 2017-09-13

**Authors:** James D Ford, Jolène Labbé, Melanie Flynn, Malcolm Araos, IHACC Research Team

**Affiliations:** 1 McGill University, Montreal, QC Canada; 2 Priestley International Centre for Climate, University of Leeds, Leeds, LS2 9JT UK

**Keywords:** Adaptation, Adaptation readiness, Arctic, Climate change, Nunavut, Inuit

## Abstract

There is limited knowledge on institutional factors constraining and enabling climate change adaptation in Arctic regions, or the overall readiness of governing bodies and communities to develop, implement, and promote adaptation. This paper examines the preparedness of different levels of government to adapt in the Canadian Arctic territory of Nunavut, drawing upon semi-structured interviews with government personnel and organizations involved in adaptation. In the Government of Nunavut, there have been notable developments around adaptation planning and examples of adaptation champions, but readiness for adaptation is challenged by a number of factors including the existence of pressing socio-economic problems, and institutional and governmental barriers. Federally, there is evidence of high-level leadership on adaptation, the creation of adaptation programs, and allocation of funds for adaptation, although the focus has been mostly on researching adaptation options as opposed to supporting actual actions or policy change. The 2016 Pan-Canadian Framework on Clean Growth and Climate Change, and increasing emphasis on climate change federally and in the Government of Nunavut, offer opportunities for advancing adaptation, but concrete steps are needed to ensure readiness is enhanced.

**Electronic supplementary material:**

The online version of this article (10.1007/s10584-017-2071-4) contains supplementary material, which is available to authorized users.

## Introduction

The Arctic climate is warming at least twice the global average, with projections indicating that Circumpolar regions will see the most rapid climate change globally (Larsen and Anisimov 2014). Impacts of climate change on sea ice conditions, permafrost, and extreme weather have already been documented and will accelerate in the future, with associated implications for northern infrastructure, food systems, subsistence livelihoods, health, and well-being (Larsen and Anisimov 2014). In light of experienced and projected climate change, adaptation has emerged as an important component of climate policy in Arctic regions, with efforts already being made to reduce vulnerability and enhance resilience across scales (Knapp and Trainor 2013; Ford et al. 2014; Ford et al. 2015; AACA 2017).

We have a growing understanding of opportunities for adaptation in the Circumpolar North, but few studies have focused on the institutional factors constraining or enabling adaptation in the Arctic, or examined the overall willingness and preparedness of governing bodies and communities to develop, implement, and promote adaptation. Responding to this gap, this paper evaluates what is being done to prepare for adaptation in Arctic Canada focusing on the territory of Nunavut, with the aim of informing efforts across scales to advance adaptation planning and identify adaptation needs. The work builds upon scholarship on *adaptation readiness* which provides a framework for evaluating the process through which adaptation is entering decision-making with respect to overarching factors critical for adaptation taking place.

## Conceptual approach

**Fig. 1:**
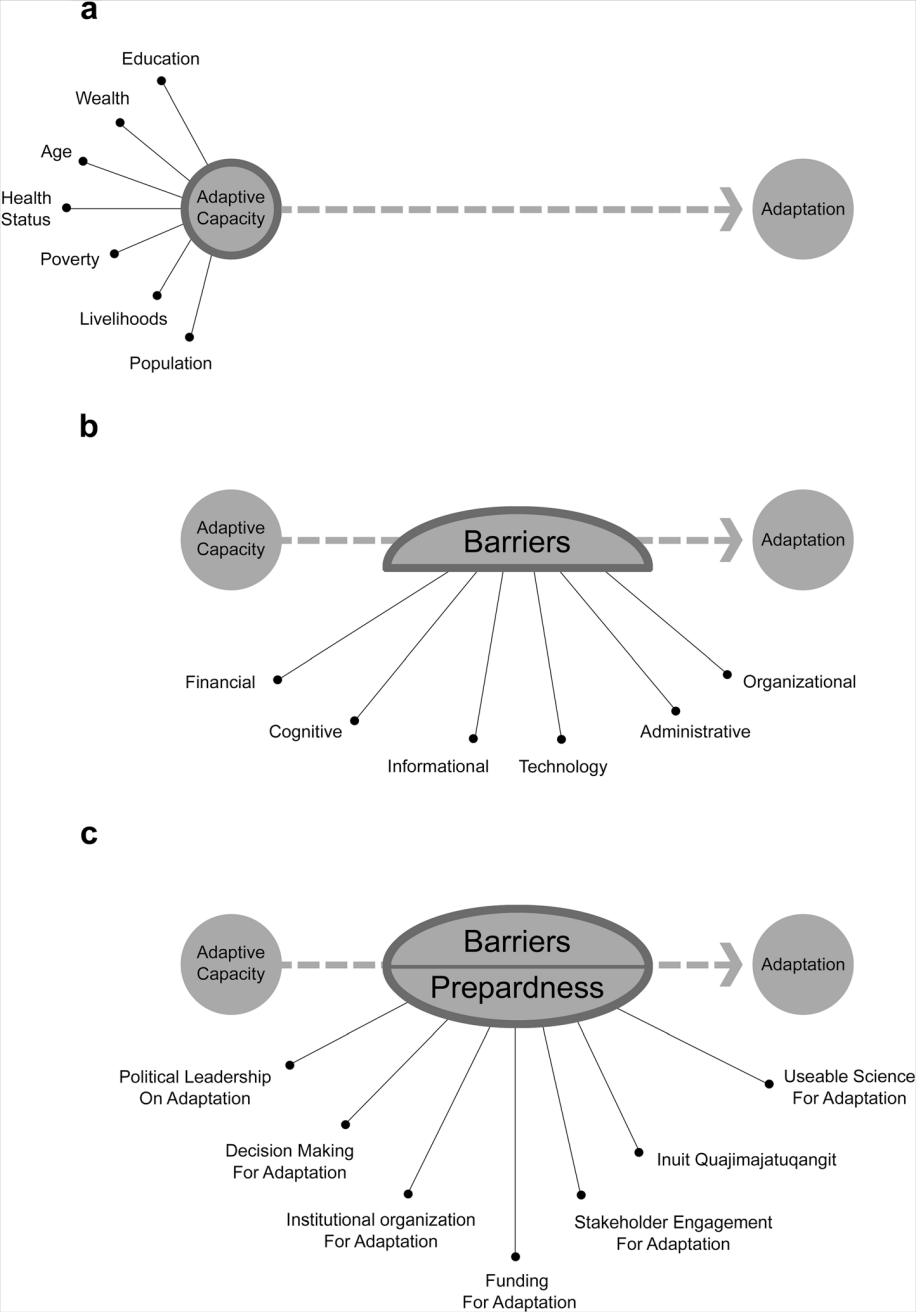
Different approaches for examining adaptation. **a** Adaptive capacity-focused assessment. **b** Barriers-focused assessment. **c** Adaptation readiness-focused assessment

Several approaches have been used in the general literature to examine the status of and needs for adaptation (Fig. 1). Adaptive capacity assessments examine the ability to adapt, focusing on a variety of social, political, economic, technological, and institutional factors (Engle 2011). Such assessments characterize what degree of change can be adapted to, but are limited by the fact that high capacity will not necessarily translate into the development and implementation of adaptation polices, programs, and actions (Adger and Barnett 2009). Other studies have focused on identifying barriers to adaptation and have examined means to overcome them (Moser and Ekstrom 2010; Eisenack et al. 2014). While insightful, these approaches have been criticized for overlooking the *process* through which adaptation occurs (Biesbroek et al. 2015). Another body of scholarship focuses on documenting and examining actual adaptation actions taking place (Chen et al. 2016; Lesnikowski et al. 2011), and has developed important baseline information on how adaptation is occurring, but does not capture the broader governance and institutional factors constraining and enabling adaptation.

We utilize an adaptation readiness-based approach. Readiness can be defined as “the state of being fully prepared for something” or “willingness to do something” (OED 2017). The concept of readiness has been widely used in business management, psychology, and education fields to understand preparedness for change, where readiness is viewed as being in a position to take advantage of change (Table 1 supplementary materials). Adaptation readiness focuses on identifying and characterizing what is actually being done to prepare for adaptation, focusing on the strength and existence of governance structures that determine the preparedness to build support for adaptation action and effectively develop, implement, and monitor adaptation interventions (Ford and King 2015).

A “readiness”-focused approach assesses the likelihood that adaptation will take place and identifies areas where intervention is needed to improve preparedness (Khan and Amelie 2015; Tilleard and Ford 2016). Readiness complements other approaches to adaptation assessment: readiness acknowledges barriers to adaptation but, more importantly, examines the processes that enable adaptation decision-making and facilitate change. A readiness approach considers underlying components of adaptive capacity, but focuses on explaining if and how these factors contribute to creating supportive institutional and governance environments for adaptation. Within the nascent adaptation readiness scholarship, research has focused on either the general policy landscape (Ford and King 2015; Salamanca and Nguyen 2016; Tilleard and Ford 2016; Araos et al. 2017), or defined readiness in terms of ability to leverage investments for adaptation (Chen et al. 2016).

We modify the general adaptation readiness framework (ARF) proposed by Ford and King (2015) for application in an Arctic context (Fig. 1, supplementary materials), integrating factors and considerations unique to adaptation in Nunavut, including feedback from decision-makers and traditional knowledge (section 3.2). The framework is used to guide data collection focusing on the Governments of Nunavut (GN) and Canada (GC). In doing so, we acknowledge the importance of adaptations undertaken by individuals and households, with our focus on institutions and planning reflecting significant challenges to adapting to the rapid changes projected for the region that require government intervention and leadership (Ford et al. 2015). The ARF is composed of seven factors critical for developing, implementing, and monitoring adaptation policies, programs, and actions, and seeks to develop broad-scale insights on readiness (see Table 2 in supplementary material).

*Political leadership* is critical for initiating the process of adaptation, which involves responding to future unknown risks where mandates, laws, and demands for action typically do not exist (Dannevig et al. 2013; Moser 2014). In Arctic regions, where resource constraints present significant challenge to decision-making, present-day policy priorities often overshadow the focus on longer term, less immediate challenges such as adaptation. Leadership is evident when governments state adaptation as a priority and take a lead on initiating and strategically directing the adaptation process (Greiving and Fleischhauer 2012; Ford and King 2015; Henstra 2016; Jude et al. 2017). It can be assessed by examining various actions such as statements from leaders (e.g., prime-minister, premier, mayor, departmental director) on the importance of adaptation, creation of national adaptation strategies, development of legal mandates to consider climate change, and/or adaptation in department or government plans.

*Decision making* for adaptation occurs under conditions of uncertainty surrounding climate change projections and in the context of multiple barriers to action, which can constrain consideration of adaptation. In the Arctic, the problem of uncertainty is exacerbated by the absence of long-term, reliable data on local climatic conditions and wide variations in the factors that affect local climatology. Adaptation decision-making is enhanced where multiple drivers and pathways creating risk and vulnerability are addressed, where there is flexibility and understanding that the adaptation process is iterative, and where robust responses are developed in the face of uncertainty (Ekstrom et al. 2017).

*Institutional organization* has an important role providing the political and administrative structure that can either enable or restrict adaptation, and while well-developed institutions may be indicative of high adaptive capacity they are not always synonymous with high readiness (Preston et al. 2011; Henstra 2016). Adaptation efforts are often most effective when a single coordinating body is responsible for overseeing and leading the process of developing and promoting adaptation, including through an interagency group or a department or branch/section within a department (Dickinson and Burton 2011). The existence of coordinating bodies responsible for overseeing adaptation efforts, including the ability to mobilize leadership and resources, develop legal and regulatory frameworks for adaptation, and plan for the short- and long-term, have also been noted as important for adaptation to occur (Bauer et al. 2012; Mukheibir et al. 2013).

*Adaptation funding* refers to specific funding and resources dedicated to adaptation efforts, and is required to cover the capital, maintenance, and human resource costs to research, and to identify, implement, and maintain adaptation actions (Moss et al. 2013). Many adaptation options will be difficult to implement with existing available funds, particularly in Nunavut where the federal and territorial government resources are already stretched to meet existing policy goals. Funding is closely connected to several other adaptation readiness components, including decision-making, where a lack of funding can act as a barrier to implementation of adaptation efforts.

*Stakeholder engagement* captures the inclusion of stakeholders and communities by government departments in adaptation planning, goals, timelines, and implementation. Bauer et al. (2012) outline how means of engagement range from informative participation, where stakeholders are kept informed and provide input into adaptation processes, to decisional participation, where stakeholders are given a say in decision-making. In the North American Arctic, there is widespread agreement that decisional engagement must underpin the development of adaptation policies, programs, and actions given the history of colonization in many regions, and top-down development of policies which historically represented outside notions of progress and well-being (McNeeley 2012; Knapp and Trainor 2013; Ford et al. 2016).

*Inuit Qaujimajatuqangit* (IQ) refers to the inclusion and integration of Inuit traditional knowledge and cultural values in adaptation efforts, which are essential to adaptation across spheres of Inuit life (Pearce et al. 2015). The GN is required to consult with Inuit on all aspects of governance, and the GN strives to operate following eight IQ guiding principles (see supplementary materials). While aspects of IQ could also be covered by stakeholder engagement, IQ goes well beyond engagement, and speaks to need for adaptations in Nunavut to be rooted in cultural values, without which adaptation could reproduce the failures of past policies (Cameron et al. 2015).

*Usable science* refers to the quality, timeliness, and pertinence of science available to inform and support adaptation decision-making (Dilling and Lemos 2011). In this paper, pertinence of science refers to the extent to which research focuses on and produces output key to decision choices. Quality refers to the amount the research is trusted and valued by decision-makers and community members, which affects the likelihood of uptake in decisions. Timeliness refers to the availability of findings according to decision-making agendas (Ford et al. 2013). The process of *how* research is conducted has particular significance in Arctic Canada where there is distrust of science based on a history of colonial research practices directed by outside interests (Castleden et al. 2012).

## Methods

### Study region

Nunavut is one of three territories in Northern Canada, created in 1999 through the Nunavut Land Claims Agreement (Fig. 2, supplementary materials). The territory has a population of 36,585 (84% Inuit), living in 26 small, remote communities. Communities are primarily fly-in, with some connections between communities available by sea-ice in winter and by boat in summer, and all but one are coastal. Nunavut residents (“Nunavummiut”) make a living through a mixed-economy consisting of waged employment and traditional subsistence practices (hunting, fishing, trapping). Communities face numerous challenges―including healthcare access, high levels of poverty, food and housing insecurity, and unemployment―owing to their unique and remote geographic location, rapid livelihood changes over the last half century, and history and ongoing experience of colonization (NTI 2014) (see supplementary materials).

### Data collection

To obtain information on the status of and progress on each of the factors in the adaptation readiness framework (ARF), we conducted in-depth semi-structured interviews (*n* = 32) with key informants in 2015 and 2016. These included decision-makers (such as an Assistant Deputy Minister (ADM), Chief Officer), various levels of policy analyst (junior to senior), program managers from the GC and GN, and staff knowledgeable about adaptation from the Nunavut Research Institute, Nunavut Tunngavik Incorporated, and other organizations working with the government (i.e., consulting firms, standards developers) (Table 3, supplementary materials). Interview questions were structured according to the ARF factors, and a question guide was created for each participant according to their position and area of expertise (see supplementary materials). In addition to interviews, our study was conducted in tandem with and drew on subsequent work by our research team characterizing the adaptation landscape in Nunavut through a systematic review of government documents on adaptation at the community, territorial, and federal levels (Labbe et al. 2017). Information obtained provided context and background to the study, and was also used to triangulate and validate the results derived from the interviews.

For the purpose of data collection, we defined adaptation consistent with Dupuis and Biesbroek's (2013, p. 1480) definition of adaptation policy, specifically “The process leading to the production of outputs in forms of activities and decisions taken by purposeful public and private actors at different administrative levels and in different sectors, which deal intentionally with climate change impacts, and whose outcomes attempt to substantially impact actor groups, sectors, or geographical areas that are vulnerable to climate change.” We acknowledge that this definition might overlook policies designed to build generic adaptive capacity or disaster risk reduction efforts, noting this focus reflects: (i) the significant threat posed by climate change to Arctic regions and necessity of targeted adaptation policies alongside efforts to build generic capacity (ITK 2016; Ford et al. 2017, in press); and (ii) the need to bound the study to provide a basis for tracking progress over time (Ford and Berrang-Ford 2016).

### Analysis

Qualitative analysis was applied to interview transcripts and notes to extract and summarize key themes and patterns across the data through a multi-step process of coding and memo creation. This process included the following: analytical memo creation, attribute and holistic coding, and is described in more detail in the supplementary materials. To increase rigor, themes, memos, and codes were discussed between two team members throughout the analysis process. All qualitative coding was performed with RQDA software. For limitations of the study, see supplementary materials.

## Results

### Political leadership

Leadership on adaptation should be evaluated in relation to roles and responsibilities for adaptation at different levels of government. The Government of Nunavut (GN), as outlined in the territorial strategic planning document on adaptation (*Upagiaqtavut*), is responsible for increasing adaptive capacity within the territory through (i) ensuring Nunavummiut are equipped with the tools, skills, and knowledge for adapting; (ii) partnership building to facilitate a coordinated approach for adapting; (iii) supporting research and monitoring of impacts; (iv) promoting education and outreach; and (v) through government policy and planning (GN 2011). Among GN interviews and in the documents reviewed, there was little evidence of leadership for adaptation at the highest levels of decision making (e.g., ADM level, Premier). There is no requirement to consider climate change or work on adaptation efforts within the GN’s current mandate, and there is little evidence to indicate that the territorial government views adaptation as a priority at the time this research was conducted. Rather, interviews described other issues such as resource development as forming the main priorities for the territorial government. Illustrative of this, *Upagiaqtavut* outlines key principles for how the GN should approach adaptation, but there is no requirement for departments to consult the document and no updates on progress towards achieving outlined objectives have been published:

“…..I don’t think there’s ever been an update on progress towards implementing Upagiaqtavut …There should be periodic, even bi-annual, annual updates on how far we've gone on meeting the goals of those pillars.” (GN employee).

Many participants further highlighted how climate change is a polarized issue in Nunavut, challenging decision-making on the issue:

“…[we have] pro-development groups saying that climate change is not happening, or that climate change is some environmentalist agenda to control us, [and other] people who say it’s real, it’s happening, we’ve got deal with it, it’s an urgent need.”

While efforts are being made to mainstream adaptation into decision-making through the GN’s Climate Change Secretariat (NCCS), which is the primary government body responsible for implementing *Upagiaqtavut*, many expressed the need for leadership at the level of the premier, ministers, and departmental ADMs.

The federal government, as outlined in the 2011 Adaptation Policy Framework (APF), is responsible for building adaptive capacity through increasing awareness of climate impacts, encouraging economic growth, establishing legislative frameworks conducive to national adaptation, and communicating climate change information (Government of Canada 2011). In December 2016, the federal government released the Pan-Canadian Framework on Clean Growth and Climate Change (PCF), with adaptation on one of the four main pillars (Government of Canada, 2016). Within the PCF, the role of the federal government to work in close collaboration with other government levels and support their actions through targeted support is reaffirmed, while also committing to give provinces and territory flexibility in moving forward with policies and actions most appropriate to their needs.

Federally, several GC interviewees discussed how the political climate and priorities set out by the previous Conservative government (2006–2015) was not always conducive to proactive leadership on climate change. Despite this, the APF was described as a high-level policy document, with the broad content of it described by GC interviews to allow for federal departments to tailor interpretations according to needs. Equally, it was noted that because the APF is not prescriptive, there is no mandate or requirement for departments to create adaptation plans or conduct risk assessments, nor is there any outlined strategy with steps towards concrete adaptation efforts. Notwithstanding, all GC respondents noted how the framework provides grounding and justification for adaptation efforts federally:

“It’s very high level, it’s somewhat vague in general and overly vague. But it’s very useful because when we’re doing something new in the department we have to show the rationale.” (GC employee).

Additionally, within many GC departments, participants reported that climate change is on the agenda of their senior-level managers and is a priority concern for internal operations since it incorporated into many business corporate risk profiles. At the time of the research, the PCF had not yet been released so we were not able to document perspectives on its potential to promote adaptation (see discussion).

### Decision-making

Awareness of climate change and the importance of adaptation was described to be increasing within departments of the GC as a result of specific funding programs, including Health Canada’s (HC) Climate Change and Health Adaptation Program, Indigenous and Northern Affairs (INAC) Climate Change Adaptation Program, Natural Resource Canada’s Adaptation Platform, and Transport Canada’s Northern Transportation Adaptation Initiative (Table 3, supplementary materials). However, while adaptation was described to be increasing on the GC agenda, outside of targeted programs, integrating climate change considerations into decision making was described as secondary in many instances.

In the GN, the incorporation of climate change and adaptation into decision-making was noted to be increasing, as well as within Inuit organizations such as Nunavut Tunngavik Incorporated, but was described to be still limited and fragmented. Many GN interviewees stated that their departments try to include climate change where possible or when it is most relevant, but explained that it is often overlooked in the face of more pressing competing priorities and short-term needs (i.e., housing crisis, food insecurity). Climate change was described to be rarely a primary consideration, even in light of dramatic warming observed in Nunavut, and a factor of limited importance in altering decisions already made:

“Whether climate change impacts a project or not will not be the deciding factor in whether a project is approved and goes through.” (GN employee).

“The biggest challenge with adaptation is that it not tangible for long-term or for planning, it’s more conceptual…how to incorporate adaptation [into] policy, it’s really tough and so I don’t think people are really thinking about it.” (GN employee).

Exceptions to the low priority given to adaptation across GN departments were noted, where impacts have directly observed financial consequences in the short-term. For instance, infrastructure was described as the area where the GN is most engaged in adaptation, reflecting sensitivity to climate impacts and significant cost implications if climate considerations are not integrated at the design stage. In the upgraded Iqaluit airport currently under construction, permafrost sensitivity and degradation was a major consideration in planning, where the location of the future taxiway was moved from the originally selected site to prevent future problems with permafrost degradation.

In other instances, interviewees reported that adaptation is sometimes occurring in response to broader changes and needs, without climate change being the main driver. An example referred to here was the construction of community wharf’s to promote economic development but also for taking advantage of more ice-free open water with warming temperatures. A few participants also cited the role of the Nunavut Land Claim Agreement and devolution as examples of adaptation, explaining the importance of ensuring protection of the environment and management of wildlife under a changing environment for Inuit use. While these examples are potentially illustrative of mainstreaming adaptation, in many instances, there was little indication either from interviewees or in reviewed documents that the impacts of climate change are being considered in these responses.

### Institutional organization

A key theme emerging across interviews concerned the importance of coordinating bodies for adaptation at both the territorial and federal levels. Within the GN, the NCCS was widely reported to have a central coordinating role for adaptation, described by a number of interviewees as an “adaptation champion” and central to driving the GN activities in this area. The Pan-Territorial Adaptation Partnership is another key organizing body for adaptation planning and implementation that was widely noted to have an important coordinating role. Through this partnership, the governments of Yukon, Northwest Territories, and Nunavut collaborated to create an adaptation strategy and work to identify and implement tangible adaptation measures. Federally, the departments who received funding under the Clean Air Agenda were highlighted as the main coordinating bodies for adaptation (Table 3, supplementary materials). Environment Canada (EC) is the lead federal department for climate change and adaptation, where they have two main roles: (i) to provide recommendations and advice to senior managers on how to advance adaptation from the federal perspective, and (ii) to provide the core fundamental scientific information about climate change to other departments and the public. INAC is the federal lead for work happening in the north, where they are seeking to assist and coordinate adaptation through engaging with territorial and other federal departments.

A number of challenges to institutional organization around adaptation across and within jurisdictions were also noted. Examples included the limited capacity of groups such as the NCCS with a small number of individuals employed and high turnover within departments creating challenges of institutional memory; concerns with certain federal departments employees not understanding the context of the north; difficulties with certain federal departments in communicating what resources are available and forthcoming (e.g., Environment Canada with climate modeling information); concerns on the restrictive participation of certain groups (e.g., comment that the Adaptation Platform is very industry focused and membership operates through invites); and restrictions on the ability to coordinate on adaptation work across departments due to a lack of political leadership (both territorial and federal).

### Funding

Over the last decade, the majority of funding for adaptation in Nunavut has been from the federal government through the Clean Air Agenda. From 2007 to 2011, $85.9 million was invested and from 2011 to 2016 $148.8 million, with at least $40–49 million allocated to adaptation in Northern Canada. The 2016 federal budget announced a total of $129.5 million towards adaptation and climate-resilient infrastructure for the next 5 years, and states that as of yet have undecided amount will go towards building resilience in the North (Government of Canada 2016). Despite this, all interviewees highlighted the deficit in the amount of funding needed for adaptation in the north broadly and Nunavut specifically. Areas described to need greater fund allocation included increased funding for communities and the GN to engage in adaptation planning and funds to support and sustain adaptation implementation at the community level. The majority of funds invested to date has focused on impact assessment and adaptation plan development, and while recognized as essential, were also noted to be only the beginning of the long-term process towards actual implementation. Concerns were also expressed that there is no specific funding or budget for climate change or adaptation within the GN budget, which some believed was a hindrance to departments’ abilities to prioritize and engage in adaptation planning and projects.

“[For]Our department it comes down to what [territorial] priorities are and what money they have left in their budget to spend on anything additional, [and] the government doesn’t set aside a budget for climate change projects.” (GN employee).

Both the GN and GC cited the uncertainty in federal funding cycles as a challenge for long-term planning on adaptation:

### Stakeholder engagement

In the context of this component of the ARF, stakeholders are referred in two ways: first, within government, understanding stakeholders to be key players in adaptation efforts, such as an entire department, a section or branch within a department, or a specific individual responsible for decision-making or coordination; second, communities in Nunavut are also considered stakeholders, particularly in light of the Nunavut Land Claim Agreement. At both the federal and territorial level, partnerships and engagement with stakeholders surrounding adaptation were reported to be often ad hoc, varying by department, and dependent on individual relationships. In the GN, for example, when departments want to work on adaptation they typically engage the NCCS. Yet, at the time of the interviews, there was no formal or informal working group or partnership across departments for adaptation efforts, which led respondents to discuss the need for increased communication across departments working on similar issues. Participants at both levels also discussed how communication and building longstanding stakeholder relationships is made difficult by poor digital infrastructure in the territory (i.e., low bandwidth), low capacity, and staff shortages, and the high turnover of staff in the GN. Consequently, interviewees explained the importance of individuals within departments and relationships:

“I think a lot of it comes down to the people in the positions as well…it’s also helpful that I have that working relationship with [them], [because they] will compile all the [relevant] work that is happening and just let us know so that we’re aware of it.” (GN employee).

Stakeholder engagement with communities through ongoing consultations was often discussed as a common form of work engagement done by the GN, particularly on wildlife management and infrastructure. Under the Nunavut Land Claim Agreement, the GN Department of Environment, Wildlife Management Division is mandated to co-manage Nunavut wildlife, where Inuit have both harvesting rights and the right to participate in decision-making on management. Based on the interviews and review of documents, adaptation does not appear to be a central component of community engagement done by most departments, however, although the NCCS has made concerted efforts to engage with communities on adaptation projects and increase awareness within the limited resources and personnel they have for this.

Stakeholder engagement at the federal level was reported by one respondent as “a good mix of ad hoc relationships…and relationships that come from a more formalized umbrella.” Despite a lack of strong leadership and interest in climate change from the previous Conservative government, interviewees often cited that cross-departmental engagement within federal departments working on adaptation to be increasing, and described being optimistic about the renewed interest in collaboration from the recently elected federal government. The main federal departments working on adaptation in Nunavut were described to have good working relationships with each other and in many cases with the GN. Federal departments engaged in adaptation efforts meet in an informal adaptation working group,

“where we brainstorm what are some issues we can look at moving forward, [ranging] from health, to transportation, to industry, to public safety. It’s… a chance to collaborate… [and] work together to plan better for whatever comes along.” (GC employee).

Over the last couple years, through the Climate Change Adaptation Program, INAC has worked with territorial governments to determine how the federal government could improve its work with the territories. Respondents spoke positively about this role, although challenges such as an overlap in mandates, missed opportunities to share information, and low uptake of adaptation on either the operational or policy side of certain departments’ work were raised.

### Inuit Qaujimajatuqangit (IQ)

Consideration and inclusion of Inuit traditional knowledge, culture, and values, or Inuit Qaujimajatuqangit (IQ), in adaptation programs and planning was highly variable, and reported as an ongoing challenge for the government. Nunavut’s *Upagiaqtavut* strategic document on adaptation focuses strongly on maintaining Inuit culture and traditional knowledge systems. For example, the framework suggests transferring IQ from elders to youth by integrating climate change and IQ into school curriculum. However, when GN employees were asked about how IQ is included in thinking around adaptation and in their departmental work more broadly, answers on what “including IQ” means were wide ranging, from incorporating the IQ principles into daily work life to co-management of land and wildlife resources, and to community consultation. Indeed, there was a general confusion on what it means to meaningfully engage with IQ in adaptation work.

A few interviewees also questioned the cultural appropriateness of adaptation, highlighting the conflicting position of formally planned adaptation efforts with traditional Inuit perspectives on the future, where planning and talking about the future, especially in a negative sense is considered inappropriate by some because it can be understood to be arrogant. As a result, those who commented on this potential difference in worldview often also stressed the importance of ensuring communities, elders, and IQ play a foundational role in adaptation measures planned and undertake,

“The level of public uptake and acceptance of those [adaptation policy or planning] documents and guidelines will be determined by Inuit notions of climate vulnerability, adaptation and by what they think of what’s being asked of them by the government.” (GN employee).

### Usable science

Nearly all interviewees reported difficulties surrounding access to and use of science for decision-making for adaptation. These challenges were reported to stem from access to data, particularly up-to-date research and climate projections; data availability in a format that is understandable and usable for decision-making; and at times, a general mistrust of research and science, especially in the case of wildlife projections under different future projection scenarios:

“Just getting the information is hard, so how can you make decisions about impacts of something on a certain type of species if you don’t even have a baseline.” (GN employee).

In some cases, specific research bodies or government departments tasked with providing science, such as ArcticNet and Environment Canada, were discussed. In these cases, participants were appreciative of the science but noted the need for improved results communication, availability, and co-production of knowledge to ensure usability for adaptation decision-making:

“There’s a lot of good work going on that doesn’t make it into the hands of people who can utilize it. I think there is a gap on the dissemination side. On really getting information out to the right people, and to discuss more clearly the delineation of departmental roles on this.” (GC employee).

## Discussion and conclusion

In this paper, we identify and evaluate how adaptation is being integrated into decision-making at different levels of government, and how actors are engaging with adaptation in Nunavut, using this as basis for characterizing readiness to adapt. We note that the insights are broad and inevitably involve some trade-off of breadth for depth, underscoring the need for research focusing on specific dimensions of adaptation readiness; such work is underdeveloped across the Arctic. In the Government of Nunavut, we found that while there were notable developments around adaptation planning and reported examples of adaptation champions, planning for implementation of adaptation measures was limited for most departments. Readiness at the territorial level has been compromised by high institutional turnover, existence of other pressing issues, and a lack of resources. Federally, there is evidence of high-level leadership on adaptation, the creation of adaptation programs, and allocation of funds for adaptation, yet most of the focus has been on researching climatic impacts and adaptation options with few examples of this translating into actual adaptation actions or changes in policy.

Political leadership is required to establish adaptation as a cross-cutting issue across government departments. Over the last decade, both the Government of Nunavut and the Government of Canada have played important roles catalyzing the emerging adaptation policy landscape, but a lack of leadership on adaptation at the highest levels of decision-making (e.g., ADM, Premier) is reflected in the fact that many actions have been ad hoc and lacked long-term commitments. Legislative and regulatory requirements for adaptation are one way to ensure a greater emphasis on adaptation, have been successfully used in other contexts (e.g., Jude et al. 2017), and could be used to ensure the Government of Nunavut’s strategic planning document on adaptation (*Upagiaqtavut*) is mainstreamed for decision making across departments. The federal role, however, will likely continue to be one of supporting provinces and territories on adaptation, as outlined in the recent Pan-Canadian Framework, involving providing information, coordination, and facilitation to draw attention to adaptation, engage public and private actors, and build support for policy objectives. Such a role has been identified as an effective approach in the context of complex, multilevel political systems like Canada’s federal system (Henstra 2017).

As part of the Pan-Canadian Framework, the federal government has committed to working closely with communities and governments to create a Northern Adaptation Strategy for Canada’s Arctic territories. This represents an important development on political leadership, and combined with recent statements by Inuit organizations on the importance of adaptation (ITK 2016), portends for a more supportive institutional and governance environment. Concrete steps will nevertheless be needed within the Northern Adaptation Strategy to advance readiness. At a general level, our work identifies multiple opportunities to increase adaptation readiness, including providing a dedicated budget for adaptation activities; greater coordination across levels of government and between government departments to ensure there is synergy between policy areas of respective jurisdictions, avoid duplication, and mainstream adaptation into ongoing policy processes; and enhancing legislative power and reporting responsibilities around adaptation strategies.

More specifically, our work highlights the important facilitative and coordinating role played by “adaptation champions” in driving adaptation forward, including removing silos between departments and increasing stakeholder engagement across scales; having the expertise to access funding, especially in departments and projects without a clear budget for adaptation; raising awareness about the impacts of climate change on a department’s mandate; creating legitimacy on the need for adaptation; bringing climate change to the forefront in meetings or decisions; and working towards mainstreaming in their everyday work. Such champions in Nunavut include individuals within organizations, or in some cases, organizations themselves (e.g., Nunavut Climate Change Secretariat), that have taken up the cause of adaptation and are pushing for its inclusion in decision-making. Champions are particularly important in resource-challenged contexts with significant competing policy priorities and high institutional turnover characteristic of many Arctic regions, and as Chapin et al. (2006) argue in the context of rural Alaska, they can also link bottom up and top-down perspectives on adaptation planning and bring continuity of engagement with communities. Supporting these champions (financially and administratively) should be a priority for advancing adaptation in the territory.

Cultural values and traditional knowledge underpin multiple components of adaptation readiness. Enhancing inclusion of Inuit Qaujimajatuqangit represents a way of ensuring community and cultural values are included in understandings of what constitutes both acceptable and successful adaptation, and needs to be central to the Northern Adaptation Strategy. Our results make clear that adaptation must be understood and undertaken within its specific social and cultural context to ensure relevancy, cultural appropriateness, and avoid maladaptation. While concern on the cultural appropriateness of formally planned adaptation is important to recognize (Bates 2007), it does not mean that Nunavummiut are against adaptation, nor that planning for future impacts should be dismissed. It does, however, implore the need for researchers, planners, and decision-makers to continually engage community leaders and elders to ensure the cultural appropriateness of adaptation planning and implementation. The important role of cultural values and traditional knowledge in underpinning community resilience to a variety of stresses is illustrated in the literature from multiple Arctic contexts and long noted by communities themselves (Pearce et al. 2015), yet as our work illustrates, there is still confusion across levels of government about what it means to “include” these knowledge systems.

In support of adaptation readiness, and development and implementation of the Northern Adaptation Strategy, the creation of a northern specific adaptation research program targeted to understanding the opportunities and challenges of adaptation, bringing together communities, decision-makers, and researchers to advance adaptation across the Canadian Arctic would considerably strengthen the integration of adaptation into decision making; promote the importance of responding to climate impacts across sectors, regions, and the public; and improve access to climate information. Such a program has been proposed by the national Inuit organization ITK in response to the lack of decision-orientated research explicitly designed to inform policy and practice for adapting. New modes of science delivery are needed to support adaptation herein, involving the co-production of knowledge, iterative interactions between researchers, communities, and decision makers, and the combination of both applied and curiosity-driven research (Ford et al. 2013; Knapp and Trainor 2013).

Finally, and more broadly for the Arctic, adaptation is emerging as a priority across scales, evident in the Arctic Council’s soon to be released Adaptation Actions for a Changing Arctic assessment, commissioned by Member States and Permanent Participants. The current state of and readiness for adaptation remains poorly understood, however, and the readiness framework used here offers one tool for examining the state of play.

## Electronic supplementary material

ESM 1 (DOCX 1696 kb)
