# Supplementary material for: Readiness for climate change adaptation in the Arctic: a case study from Nunavut, Canada
Source: Clim Change. 2017 Sep 13;145(1):85–100. doi: 10.1007/s10584-017-2071-4 (PMC6959400; doi:10.1007/s10584-017-2071-4)
Supplement: Supplementary file 1 — (DOCX 1696 kb) [file 10584_2017_2071_MOESM1_ESM.docx]

**Supplementary Materials**

**Nunavut governance**

The federal government has a substantial role in Nunavut with delegate powers given to the territorial Legislative Assembly under the authority of the Parliament of Canada. Since the 1970s, responsibility for various services such as health, education, social services, housing, airports, and language has been transferred to the territorial government (Government of Canada, 2016b). The federal government provides a substantial portion of funding to the territory through a transfer program, where the Government of Nunavut (GN) then delivers services. Land and resource management in the territory are currently held by the federal government within the department of Indigenous and Northern Affairs Canada (Government of Canada, 2016b); this excludes areas held by Nunavut Inuit as part of the NLCA, where Inuit “have the right to participate in decision-making concerning the use, management and conservation of land, water and resources, including those offshore” (Nunavut Tungngavik Incorporated and Indigenous and Northern Affairs Canada, 2010, 1).

**Analysis**

All interview transcripts and notes were subjected to qualitative analysis to determine key themes and patterns across the data through a process of coding and memo creation. In order to increase rigour, themes, memos, and codes were discussed between two team members throughout the analysis process. The coding methodology is based on Auerbach and Silverstein’s (2003) methodology for coding and included three steps. All qualitative coding was performed in RQDA software.

***Step 1: Analytical memo creation***

We used analytic memos as a way to initially process and categorize information about the research process, participants involved in interviews, preliminary findings and themes, and development of a coding strategy (Saldaña, 2013). This involved a day-long workshop discussing the interview transcripts and observations between research team members. Discussions focused on applying preliminary results from the interviews to the factors outlined in the readiness framework.

***Step 2: Attribute coding***

We used attribute coding to define the key characteristics of the data to allow for future management and reference. This form of coding typically provides basic descriptive information about the transcripts (Saldaña, 2013). Two forms of attribute coding were used here. First, interview transcripts were coded according to scale: 1) National, 2) Territorial, or 3) Community. This allowed for multiscale considerations in the results. Secondly, transcripts were coded according to scale: territorial or national. Data was categorized according to scale in order to differentiate across scales and show connections, barriers across and between them.

***Step 3: Holistic coding***

We used holistic coding to categorize our data into broader project categories (Saldaña, 2013). We based broad concepts on theoretical constructs discussed in the initial analytical memo session, which we linked to the adaptation readiness framework factors. We developed a specific code for each individual factor, as well as cross cutting codes, which were applied through transcript content analysis. All coded data for each individual readiness factor code category was extracted and further analyzed within its category to understand the linkages, barriers, and sub-themes discussed within each factor. Guided by trends emerging from interview coding, a qualitative interpretation of the data was performed; this allowed for narrative creation of the adaptation readiness of multiple levels of governance in Nunavut.

**Interview guide**

The following base interview guide was modified for each participant according to their department and role

**Adaptation Broadly**

1. Can you tell me about your role as (insert role or job specific to person being interviewed) or area of department’s work more broadly?
2. Can you tell me about how your organization/department/job is involved with climate change and adaptation work?
   1. Give a definition of adaptation if needed
   2. Give some examples relating to the department or organization if I have any

**Institutional Organization**

1. Generally, how willing is your department/organization to include CC in your work?
2. Who is involved or responsible for the different phases of an adaptation initiative?
   1. Ex: planning, coordination, implementation, monitoring, evaluation etc.?
3. What regions/sectors is your institution concerned with or working in?
4. Are there any linking mechanisms (like partnerships) between your institution and other institutions involved in cc and adaptation? Between projects your institution is involved in?
   1. Probe about government partnership and private-sector or NGOs partnerships
   2. Could give some examples if needed
5. Are there interlinkages between adaptation-focused work and other projects/focuses your institution does?
6. Is there long-term planning for adaptation within your department?
7. Are there any challenges with institutional organization or operating procedures that act as barriers for adaptation initiatives? How are these currently managed?

**Funding**

1. Can you explain to me how adaptation in your organization are funded?
2. Does this work well? Are there any suggestions for improvements?

**Leadership**

1. Are there any people or organizations who are particularly important in ensuring climate change and or adaptation is incorporated into the work your organization/department does?
2. Are there any policies and/or mandates in place that ensure CC and/or CC and adaptation will be considered?

**Decision-making and stakeholder engagement**

1. Can you tell me about how decisions are made with regard to cc and adaptation in your organization/institution?
2. Once a decision has been made or implemented, how flexible is it to change or make decisions surrounding this policy/project/initiative?
3. What role does stakeholder engagement play in adaptation planning, development, implementation, and evaluation?
4. How is uncertainty accounted for in the planning and decision-making processes?
5. How is potential for maladaptation considered or accounted for in planning, and implementation decision-making processes?
   1. When planning for adaptation, how are current and future climate scenarios and impacts taken into account? *applies to both 15 & 16

**Public Support**

1. Where do you see the role of public support for adaptation and CC being?
2. How do you engage with communities? How better could you engage with communities?

**Usable Science**

1. What information or research informs adaptation policies, plans, programs, actions?
2. When cc and/or adaptation research or information collected by other institutions is shared with you, is it usable or useful for your institution and decision-making process?
3. Where and with whom do you share information or knowledge produced by your institution?

**Inuit Qaujimajatuqangit (IQ)**

1. How is IQ incorporated into your institution/department/organizations adaptation planning/development/implementation?
2. What are the benefits or TK? Are there challenges in accessing or using TK that lead to barriers in adaptation? How are these managed?

**General closing questions**

1. How do you think your department is doing with regard to adaptation in general?
   1. What are its main strengths? Areas for improvement?
   2. Can you identify any specific needs or gaps?
2. Same question for Nunavut as a whole (only for those who might know about this)

**Methodological challenges and limitations**

There are a number of challenges and limitations to our work. The vastness of Nunavut (>2m km^2^), cost of transportation between communities, and communication challenges prevented us from being able to interview municipal/hamlet governments, and restricted in-person interviews to the territorial capital, Iqaluit. Additionally, these factors meant that whilst communication throughout the research was ongoing, not all stakeholders could be consulted in-person or by phone at all stages of the research process. To manage this, close contact was kept with key coordinating stakeholder (Department of Environment Climate Change Section and the Nunavut Research Institute), including three face-to-face meetings, a number of phone meetings and abundant email communication. Further, although we attempted to include all territorial government department and all federal government departments working in the north, a small number of departments were not able to participate. These challenges need to be considered when interpreting the results.

We also note that the aim of the work was develop a general understanding of the adaptation landscape in Nunavut. The adaptation readiness framework directed us to specific factors essential for adaptation to focus on, and develop an overarching understanding on readiness. Yet we acknowledge each factor in and of itself could have been the focus of an entire research project, in which some degree of generalization and a trade-off between breadth and depth had to be made. Such trade-offs are a common challenge for studies aiming to develop broad scale insights.

**IQ guiding principles**

1) Inuuqatigiitsiarniq: respecting others, relationships and caring for people; 2) Tuunganarniq: fostering good spritis by being open, welcoming and inclusive; 3) Pijitsirniq: serving and providing for family and/or community; 4) Asjiiqatigiinniq: decision-making through discussion and consensus; 5) Pilimmaksarniq/Pijariuqsarniq: development of skills through observation, mentoring, practice and effort; 6) Piliriqatigiinniq/Ikajuqtigiinniq: working together for a common purpose; 7) Qanuqtuurniq: being innovative and resourceful; and 8) Avatittinnik Kamatsiarniq: respect and care for the land, animals and the environment

**Figures and Tables**

**Figure 1.** Modified Ford and King (2015) Adaptation Readiness Framework


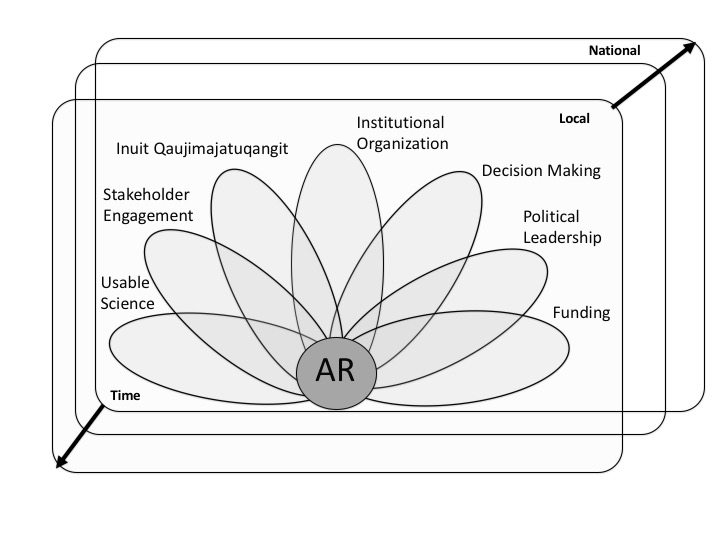


**Figure 2.** Map of Nunavut, Canada. Community list: (1) Iqaluit, (2) Pangnirtung, (3) Qikiqtarjuaq, (4) Clyde River, (5) Pond Inlet, (6) Arctic Bay, (7) Cape Dorset, (8) Kimmirut, (9) Igloolik, (10) Hall Beach, (11) Repulse Bay, (12) Coral Harbour, (13) Baker Lake, (14) Chesterfield Inlet, (15) Rankin Inlet, (16) Whale Cove, (17) Arviat, (18) Bathurst Inlet, (19) Kugluktuk, (20) Cambridge Bay, (21) Gjoa Haven, (22) Taloyoak, (23) Kugaaruk, (24) Resolute Bay, (25) Grise Fjord, (26) Sanikiluaq. The star denotes the capital city, Iqaluit. Map base data: GADM database of global administrative areas, adaptation data: author’s own.


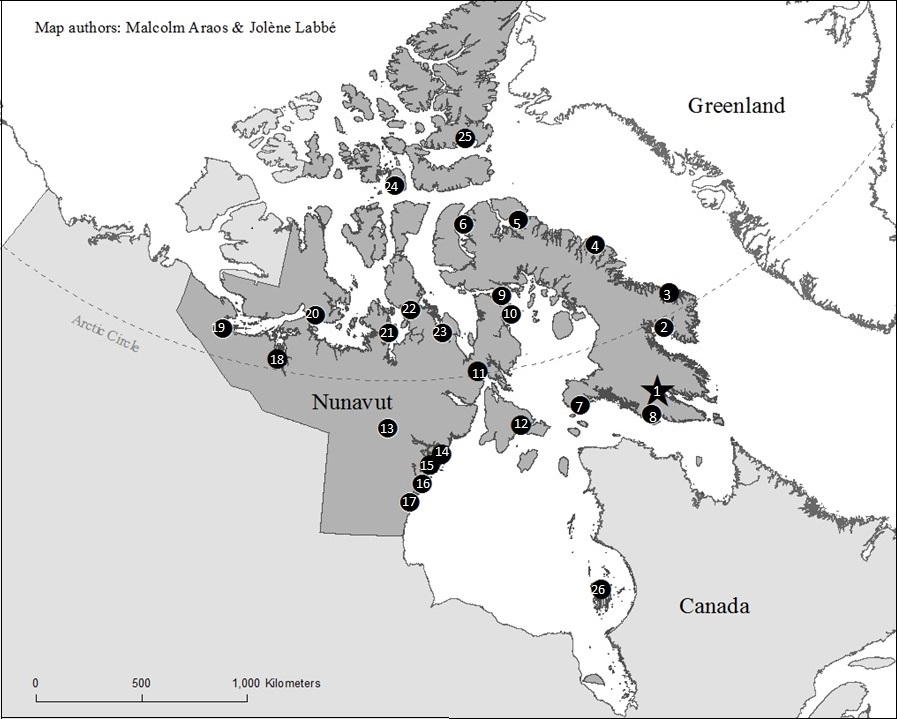


Table 1: The concept of ‘readiness’ as is used and defined across select disciplines

| **Readiness across disciplines** | **Definitions used in the literature** | **Key focus and contribution** |
| --- | --- | --- |
| Climate change vulnerability & adaptation | “*Readiness* measures a country's ability to leverage investments and convert them to adaptation actions” (Chen et al., 2015).  “Economic *readiness* captures the ability of a country's business environment to accept investment that could be applied to adaptation that reduces vulnerability (reduces sensitivity and improves adaptive capacity)” (Chen et al., 2015).  “Governance *readiness* captures the institutional factors that enhance application of investment for adaptation” (Chen et al., 2015).  “Social *readiness* captures the factors such as social inequality, infrastructure, education and innovation, that enhance the mobility of investment and promote adaptation actions” (Chen et al., 2015).  Individual level “*readiness* to engage with climate change” refers to the psychological perception that climate change is a personally relevant threat (Brügger, Dessai, Devine-Wright, Morton, & Pidgeon, 2015).  “The economic stage of development, financial and human capacity, and people’s awareness of climate change issues” have been listed as factors defining local government *readiness* to respond to environmental challenges.  “*Readiness* refers the extent to which human systems are prepared to adapt, providing an indication or measure of the likelihood of adaptation taking place” (Ford & King, 2015). | Readiness involves the ability to convert human, financial, and technical capacity into adaptation.  Readiness focuses on the supportive measures that already exist for adaptation planning.  Readiness involves the factors that enhance the application of adaptation investment.  Readiness involves the supportive institutional and governance environment specifically for adaptation. |
| Management and organizational behaviour | “Organizational *readiness* for change refers to organizational members’ change commitment and self-efficacy to implement organizational change” (Weiner, 2009).  “*Readiness* involves a demonstrable need for change, a sense of one’s ability to successfully accomplish change (self-efficacy) and an opportunity to participate in the change process.”  “The notion of *readiness* for change can be defined as the extent to which employees hold positive views about the need for organizational change (i.e. change acceptance), as well as the extent to which employees believe that such changes are likely to have positive implications for themselves and the wider organization” (Jones, Jimmieson, & Griffiths, 2005).  “*Readiness* for change is the extent to which an individual or individuals are cognitively and emotionally inclined to accept, embrace, and adopt a particular plan to purposefully alter the status quo” (Holt, Armenakis, Feild, & Harris, 2007). | Readiness involves a belief that change is needed.  Readiness involves self-belief and self-confidence about the capacity of the individual or organization’s ability to change.  Readiness involves the belief that change is the appropriate and necessary response.  Readiness involves holding a favourable view of the products of change, i.e. that change will bring positive outcomes.  Readiness involves being in a position to take advantage of opportunities for change. |
| Psychology | - “Change *readiness* reflects the process wherein people, influenced by information received from change drivers, peers, and other contextual clues, perceive the change as necessary and achievable” (McKay, Kuntz, & Naswall, 2013). | - Readiness involves a set of factors: external drivers, input from peers, and other contextual clues influence the perception that change is achievable and needed. |
| Education | - In education, *readiness* to learn refers to “the circumstances under which a learner tends to welcome or reject new information” (Knowles, Holton III, & Swanson, 2014). - People are *ready* to learn when their life situation creates a need to know something (Knowles et al., 2014). - The developmental/informational needs of people are the most important determinant of *readiness* to learn what is being taught (Knowles et al., 2014). | - Readiness involves a combination of a *need* for change with a *willingness* to change. |

Table 2: Adaptation readiness factors

| **Adaptation readiness framework factors** | **Supporting text and sources from adaptation literature** |
| --- | --- |
| Political leadership for adaptation | - Political leadership is critical for initiating the process of adaptation; leaders provide strategic direction and convince others of the need to act (Eisenack et al., 2014). - local adaptation “champions” or “entrepreneurs” are well-established in the literature as individuals that are instrumental in motivating institutional change. They leverage their positions and resources to achieve desired adaptation-related outcomes (Carmin, Anguelovski, & Roberts, 2012). |
| Institutional organization for adaptation | Clear institutional arrangements and demarcated mandates have been noted as important enablers for adaptation planning (Eisenack et al., 2014).  Clarity about who is responsible for planning adaptation is reported by policymakers as important for action (Araos, Ford, Berrang-Ford, Biesbroek, & Moser, 2016).  Burch (2010) found that centralizing adaptation planning in the Mayor’s office was an effective way to spur the implementation of adaptation regulations and legislation in three B.C. municipalities (Burch, 2010).  Eisenack et al. (2014) cite the diffusion of adaptation planning responsibilities into 52 separate municipalities in Santiago, Chile, as a barrier for city-wide coherent adaptation initiatives (Eisenack et al., 2014). |
| Availability of funding for adaptation | The lack of sustained multi-year funding has been noted by UK local authorities as a barrier for the long-term adaptation planning (Urwin & Jordan, 2008) cited in .  A case study of adaptation barriers in Sidney, Australia, found that adaptation planning competes for funding with other more “immediate issues” with which to contend (Measham et al., 2011). |
| Public support for adaptation | For example, recent research on managed retreat as a response to climate risk finds that if citizens are supportive of retreat, then the reduced conflict between citizens and the implementing entity will enable quicker retreat action (Hino, Field, & Mach, 2017). |
| Engagement of stakeholders for adaptation | The absence of integration of citizen concerns suggests that the needs of marginalized and the most vulnerable groups may not be adequately included in plans (Shi et al., 2016). |
| Interaction between science and policy for adaptation | Mechanisms for translating traditional science into tailored information for policy-makers, such as boundary organizations, are important to ensure scientific knowledge feeds into adaptation planning (Hoppe & Wesselink, 2014). |

Table 3. List and descriptions of government of Canada climate change adaptation programming relevant to Northern Canada funded under the Clean Air Agenda

| **Government of Canada Department** | **Federal program** | **Description of program** | **Funding received** |
| --- | --- | --- | --- |
| **Health Canada (HC)** | Climate Change and Health Adaptation Program for Northern First Nations and Inuit Communities | Funded 47 community-led research projects addressing the health impacts of climate change in Inuit regions (11 in Inuvialuit, 21 in Nunavut, 4 in Nunavik, and 11 in Nunatsiavut), where funds went directly to community organizations who worked with various partners to complete project goals | $17 million from 2008-2016  ~$5.6 million went to the 47 projects in Inuit Nunangat |
| **Transport Canada (TC)** | Northern Transportation Adaptation Initiative | Program that supported adaptation research and collaborative initiatives with territorial governments and not-for-profit private sector companies | Received $11 million from 2011-2016.  Lack of information on specific amount that went to work in Inuit Nunangat. |
| **Indigenous and Northern Affairs Canada (INAC)** | Climate Change Adaptation Program | Funded northern community and regional government work focused on infrastructure vulnerability; coastal erosion; sea level rise and ice dynamics; drinking water quality and availability/waste water management; extreme weather events; winter roads; and permafrost degradation. | $14 million from 2008-2011 and $20.2 million from 2011-2016,  ~$3.3 million of which went to projects in Inuit Nunangat between 2008-2011 and ~$4.8 million between 2011-2016 |
| **Public Health Agency of Canada (PHAC)** | Preventative Public Health Systems and Adaptation to a Changing Climate Program | Inuit-relevant projects funded through this program included an assessment of the burden of acute gastrointestinal illness (AGI) and adaptation to climate change in the Canadian North in selected Inuit communities (e.g. Iqaluit, Rigolet) to aid development of adaptation strategies. | Received $12 million between 2011-2016.  Lack of information on specific amount that went to work in Inuit Nunangat. |
| **Natural Resources Canada (NRCan)** | Does not have northern specific standalone climate change adaptation program, but supports adaptation efforts in Inuit Nunangat through the Climate Change Geosciences and Adaptation Program (CCGP) (2005-2016), the Adaptation Platform’s Northern Working Group (ongoing), and through Regional Adaptation Collaboratives (RAC) (ongoing) | CCGP focuses on providing scientific research to help land-use planners, industry, and regulators decrease risk and adapt northern resource development and does not report inclusion of IQ.  Northern Working Group brings together northern stakeholders to identify adaptation priorities and work to mainstream adaptation needs.  RACs are a cost-sharing programs between the federal and provincial/territorial governments, which supports adaptation planning, mainstreaming adaptation into decision-making, and promoting collaboration across stakeholders from regional to local levels | Received $35 million for adaptation related work through the *Enhancing Competitiveness in a Changing Climate* from 2011-2016.  Lack of information on specific amount that went to work in Inuit Nunangat. |
| **Fisheries and Oceans Canada (DFO)** | Aquatic Climate Change Adaptation Services Program (ACCASP) | The ACCASP included an assessment of climate change risks and vulnerabilities in the Canadian Arctic Ocean Basin, and research that support applied adaptation Tools to help mainstream climate change into departmental programs and policies in the north | Received $16.55 million in funding for 2011-2016.  Lack of information on specific amount that went to work in Inuit Nunangat. |
